# *Tatumella ptyseos* septicaemia in a tertiary hospital in Nigeria: a case report

**DOI:** 10.11604/pamj.2021.39.6.25490

**Published:** 2021-05-03

**Authors:** Atana Uket Ewa, Ernest Afu Ochang, Glory Ekpo Bassey, Bassey Ewa Ekeng

**Affiliations:** 1 Department of Paediatrics, University of Calabar, Calabar, Nigeria,; 2 Department of Medical Microbiology and Parasitology, University of Calabar, Calabar, Nigeria,; 3 Department of Medical Microbiology and Parasitology, University of Calabar Teaching Hospital, Calabar, Nigeria

**Keywords:** Septicaemia, bronchopneumonia, blood culture, case report

## Abstract

Tatumella ptyseos septicaemia in humans is yet to be reported in Nigeria with very few cases reported worldwide. This case report describes the clinical and distinctive biochemical characteristics of Tatumella ptyseos, its antibiotic sensitivity pattern and risk factors associated with Tatumella ptyseos septicaemia. Our case is a 2 months old ex-premature female from Calabar, admitted in the month of May, 2018 into the Children´s Emergency Room, of the University of Calabar Teaching Hospital, Nigeria. She presented with cough of one month and fever of three weeks, and was found to be acutely ill looking, febrile with temperature of 38.6°C, mildly pale, dyspnoeic and tachypnoeic with SPO_2_ of 80% in room air, tender hepatomegaly of 6cm and a splenomegaly of 6cm. Blood culture yielded Gram negative rods identified as Tatumella ptyseos by OXOID MICROBACT™ GNB identification kit.

## Introduction

The name Tatumella is derived from Harvey Tatum, a CDC microbiologist, and ptyseos, meaning epithet of sputum [1, 2]. *T. ptyseos*, is a member of the Enterobacteriaceae family first identified by Hollis *et al*. from strains previously called CDC group EF-9 [1-4]. Table 1 summarizes its biochemical characteristics. Six different species from Tatumella genus have been described: *T. ptyseos* and *T. saanichensis* recovered from humans; *T. citrea, T. punctata, T. terrae*, and *T. morbirosei* originally recovered from fruit [5], with *T. ptyseos* identified as a causative agent of pink disease in pineapple, contrary to popular beliefs that Pantoea citrea was the cause [6]. *T. ptyseos* and *T. citrea*are known to cause infections in humans [5]. *T. ptyseos* has been isolated from human and animal materials such as blood, sputum, stool, urine, abdominal tumour, and venous catheter [5] and in Nigeria, has been identified in fresh beef [7].

**Table 1:** biochemical reactions of *T. ptyseos* [3]

Test	Reaction/Result
Gram stain	Gram-negative rod
Triple Sugar Iron agar	Acid (yellow) slant/ Acid (yellow) butt/ No gas/ No H2S
Catalase	+ (weak)
Oxidase	-
Motility (25˚C)	+
Motility (37˚C)	-
Simmons Citrate´s (25˚C)	+
Simmons Citrate´s (37˚C)	-
Nitrate reduction	-
Gas from glucose	-
Acid from glucose	+
Lactose	-
Arabinose	+
Sucrose	+
Mannitol	-
Xylose	+
Sorbitol	-
Raffinose	-
Malonate	-
Adonitol	-
Rhamnose	-
Inositol	-
Indole	-
Methyl Red (MR)	-
Voges Proskauer (VP)	-
Urease	-
Lysine decarboxylase	-
Ornithine decarboxylase	-
Arginine dehydrolase	-
Phenylalanine deaminase	+ (weak)
Esculin hydrolysis	-
Gelatine hydrolysis	-

*T. ptyseos* has been reported to be associated with severe neonatal sepsis especially in children being fed with contaminated powdered infant formula milk [3]. All over the world, confirmed human infections with *T. ptyseos* are very rare, with less than 10 cases reported [5]. Two severe cases of *T. ptyseos* human infection in an adult and in an elderly have been reported in Brazil, a case report in an adult in Switzerland, a case report in an adult in Turkey and in a 6-day old in Malaysia [1, 5, 7, 8]. We report the first case of infection with *T. ptyseos* in a patient from Nigeria.

## Patient and observation

A 2 months old child admitted in the month of May, 2018 into the children emergency room, of the University of Calabar Teaching Hospital with complaints of cough of one-month duration and fever of three weeks duration. Cough was distressing and worse at night. Fever was high grade, intermittent and temporarily relieved by administration of paracetamol syrup. Pregnancy was booked at a gestational age of 13 weeks. Delivery was at a gestational age of 28 weeks via emergency lower section caesarean section. Birth weight was 1.65kg. Child was admitted into special care baby unit and managed for prematurity, neonatal sepsis and neonatal jaundice on account of antepartum haemorrhage. She had blood transfusion but no exchange blood transfusion and was discharged at a weight of 1.8kg having spent a month in special care baby unit. Child was fed with infant formula and breast milk from birth. She is the second child in a monogamous setting and has a 7-year-old male sibling. The father is a civil servant while the mother is a businesswoman.

Physical examination showed an acutely ill looking child, febrile with temperature of 38.6°C, mildly pale, dyspnoeic and tachypnoeic, with a weight of 3.7kg (3^rd^ percentile), length of 50cm (below the 3^rd^ percentile) and occipito frontal circumference - 38cm (z-score). Respiratory rate was 66cycles/minute, with SPO_2_ of 80% in room air. The heart rate was 186 beats/minutes with normal heart sounds. The abdomen had a tender hepatomegaly of 6cm and a splenomegaly of 6cm. Liver was 8cm below the right costal margin, soft and smooth. Spleen was 2cm below the left costal margin, and non-tender.

**Figure 1:**
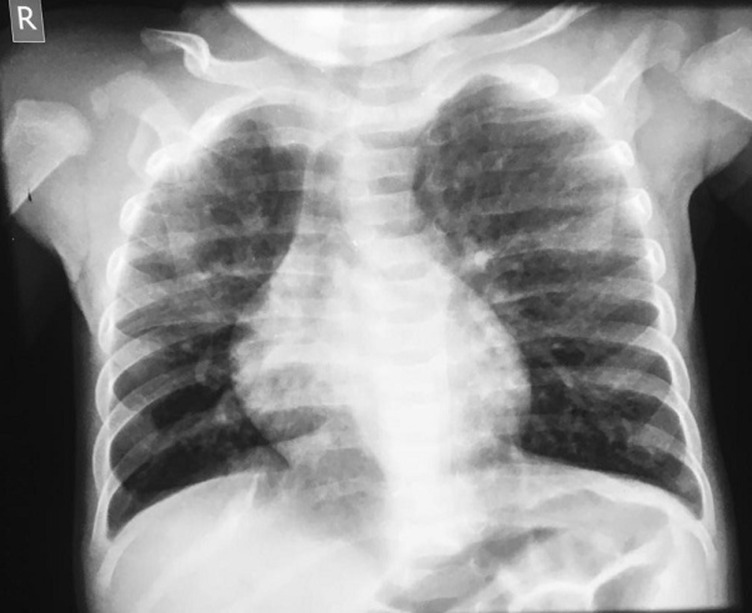
chest X-ray findings in a 2 months old ex-premature female child with *T. ptyseos* septicaemia

Laboratory findings showed a PCV of 21%, random blood sugar of 4.7mmol/L, HIV screening was negative, Full blood count; Total WBC of 12.6 x 10^9^/L, Neutrophils: 65%, Lymphocytes: 33%, Platelets: 17^9^x10^9^/L, Malaria parasite test: positive, Gene Xpert: negative, Mantoux test: 0 mm, Chest X-ray showed hazy opacities on the right upper and middle lung lobes, with left upper lobar opacities and pneumatoceles (Figure 1). Blood culture yielded *T. ptyseos*. Patient was on oxygen at 2L/min and was transfused with 60mls of settled cells. Post transfusion PCV was 25%. While on admission, patient received IV Ceftriaxone and IV gentamicin and spent 5 days during which fever subsided and respiratory rate reduced, and was thereafter discharged. One week later, patient represented and was admitted on account of the same but worsening symptoms. A diagnosis of Septicaemia with heart failure was made to rule out bronchopulmonary dysplasia and pulmonary tuberculosis. Empirical antibiotic (IV Ceftriaxone, IV Gentamicin, Suspension Azithromax) therapy were commenced. IV Furosemide and tablets digoxin was also given. On the second day of admission, IV cloxacillin was commenced, as the child remained ill and IV gentamicin discontinued. 3 days into admission, child developed seizures which was generalized tonic clonic lasting about 1-2 minutes. She had two episodes. The second episode was aborted with IV diazepam. The random blood sugar at the time was 2.9mmol/L. SPO_2_ was 67%. Intravenous fluid was commenced at 60% maintenance and phenobarbitone was also commenced at maintenance dose. She received blood transfusion, Nil per oral and cautious intravenous fluid, and oxygen therapy. Frusemide and digoxin were also added. Despite these measures, she was still very ill and in severe respiratory distress. CSF analysis was not in keeping with meningitis. She remained very ill, had intermittent hypothermia with subnormal temperature and died 5 days after admission. Blood culture result of sample taken on the third day of admission and received 3 days after patient´s demise yielded growth of *T. ptyseos*. Antimicrobial susceptibility test result showed sensitivity to Amikacin, intermediate to Ceftazidime and Ciprofloxacin, and resistant to Ceftriaxone, Cefuroxime, Gentamicin, Amoxicillin-Clavulanate, Cefepime, Cefoxitin and Colistin.

## Discussion

**Figure 2:**
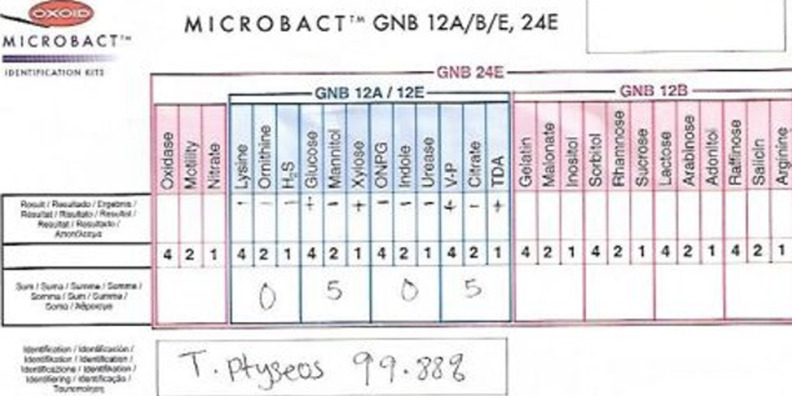
identification of *Tatumella ptyseos* by OXOID MICROBACT™ GNB identification kit

Blood culture was done using brain heart infusion broth and thioglycolate broth, and aerobically incubated. Both broths when subcultured in 5% sheep blood agar and MacConkey agar, yielded growth of smooth, convexed and non-lactose fermenting colonies measuring approximately 1-1.5 mm in widest diameter after incubating at a temperature of 35°C for over 48 hours. Gram negative rods were observed by light microscopy and were identified by the OXOID MICROBACT™ GNB identification kit as *T. ptyseos* (Figure 2). Antimicrobial susceptibility testing was done using the Clinical Laboratory Standard Institute guidelines for Enterobacteriaceae group of organisms [9]. The risk factors in our patient were preterm delivery, low birth weight and the use of infant formula milk. Reported risk factors include older and younger age especially pre-term and low birth weight neonates, the use of feeding tubes in neuromuscular disease patients, diabetes mellitus and immunosuppression [1, 3]. Jalal M *et al*. (2014) reported *T. ptyseos* strains isolated from powdered infant formula milk consumed in a neonatal intensive care unit in Iran [3]. This case may have acquired this organism through infant formula milk. Proper hygienic conditions should be maintained during the processing and preparation process and in the handling of raw milk and milk products. Our patient had septicaemia with pneumonia in heart failure. Few cases of *T. ptyseos* septicaemia have been documented in medical literature [1, 5, 7, 8]. *T. ptyseos* has also been implicated in clinical conditions like pneumonitis, asthmatic bronchitis, pharyngitis, Wegener granulomatosis, pneumonia, chronic lung disease, pulmonary oedema, pulmonary tuberculosis, and gastrointestinal infection [1, 3, 10].

The identified organism had the unique biochemical characteristics of *T. ptyseos* as described. It shares many properties of the Enterobacteriaceae family, but with few differences with respect to biochemical characteristics [1-4]. The antibiotics used for the patient´s empirical therapy wasn´t sensitive to *T. ptyseos*. The inability to do an early blood culture for our patient was a major limitation to her clinical management and may have contributed to the poor outcome of this case. And when it was done, the *T. ptyseos* isolates were identified by the OXOID MICROBACT™ GNB identification kit, which is not routinely available in the developing countries.

## Conclusion

*T. ptyseos* septicaemia in humans has not been reported in Nigeria and is scarcely reported globally. Prompt investigation especially cultures of appropriate specimens and identification kits will aid timely diagnosis and therapeutic intervention.
